# The Effect of Endoscopic Bariatric and Metabolic Therapies on Gastroesophageal Reflux Disease

**DOI:** 10.3390/medicina57080737

**Published:** 2021-07-21

**Authors:** Su-Young Kim

**Affiliations:** Division of Gastroenterology, Department of Internal Medicine, Yonsei University Wonju College of Medicine, 20 Ilsan-ro, Wonju 26426, Korea; breeze1212@yonsei.ac.kr; Tel.: +82-33-741-0507; Fax: +82-33-747-3538

**Keywords:** endoscopic bariatric and metabolic therapies, intragastric balloon, endoscopic sleeve gastroplasty, gastroesophageal reflux disease, obesity

## Abstract

Obesity is a chronic disease that is becoming increasingly more prevalent and is associated with many health problems, such as metabolic syndrome. The treatment options for obese patients include lifestyle modification, medications, endoscopic bariatric and metabolic therapies (EBMTs), and surgery. In particular, EBMTs have an excellent therapeutic effect and are less invasive than bariatric surgery. Although it is clear that EBMTs are relatively safe procedures, they can result in several adverse events. Among them, the relationship between EBMTs and gastroesophageal reflux disease (GERD) is unclear. Several studies have demonstrated that an intragastric balloon (IGB) may worsen GERD. There are a few studies on the effects of endoscopic sleeve gastroplasty (ESG) on GERD, but the linking evidence is insufficient. However, the conclusion is not simple. Because obesity is an important cause of GERD, and GERD naturally improves with weight loss after EBMTs, it is not easy to evaluate accurately the effect of EBMTs on GERD. This review aimed to discuss the effect of EBMTs on GERD and suggest future research directions.

## 1. Introduction

Obesity is a complex metabolic disease associated with many health problems, including diabetes mellitus, hypertension, cardiovascular disease, obstructive sleep apnea, malignancy, and gastroesophageal reflux disease (GERD) [1,2,3]. Obesity is not a single disease but is accompanied by a variety of comorbidities, so the increase in the obese population at the national level has caused a significant increase in medical expenses [4]. Therefore, resolving obesity is an important issue from the national health and medical perspectives. The current obesity treatments rely on lifestyle modifications, diet control, exercise, and medication, but these methods often do not result in effective weight loss, and persistence is a problem even if there is a weight-loss effect [5,6]. Therefore, improved treatment methods are needed to solve these problems.

Bariatric surgery is performed to treat extreme obesity and metabolic diseases accompanying obesity and includes adjustable gastric banding, laparoscopic sleeve gastrectomy (LSG), and Roux-en-Y gastric bypass (RYGB) [7]. Bariatric surgery has good effects, but patients do not choose this treatment due to its high cost and permanent resection of the gastrointestinal tract [8]. Research and development on endoscopic bariatric and metabolic therapies (EBMTs) have been conducted to obtain an effect similar to that of bariatric surgery [9]. EBMTs are expected to have a comparable effect to that of bariatric surgery and are superior to surgery in terms of cost and safety, therefore they are relatively easily accessible. Representative EBMT methods include the intragastric balloon (IGB), which artificially reduces stomach volume by inserting a balloon, and endoscopic sleeve gastroplasty (ESG), which is a gastric reduction procedure through an endoscope [10,11].

EBMT reduces weight and ameliorates obesity-associated complications [12,13]. However, EBMTs can result in adverse events, including nausea, abdominal pain, and gastroesophageal reflux disease (GERD) [12,13]. GERD may diminish when EBMT is performed on obese patients because GERD may occur due to obesity, but GERD may also worsen due to the EBMT even though the patient has lost weight. Recent studies have suggested that anatomical changes associated with bariatric surgery (LSG and vertical gastric banding) may increase new-onset GERD in asymptomatic patients [14,15]. However, the mechanism of EBMT in GERD is unclear, and only small studies have indicated that anatomical changes associated with EBMT may compound GERD. As the obese population increases, the targets of EBMT are gradually increasing. Therefore, managing the complications related to the procedure is emerging as an important issue. In particular, GERD can significantly reduce the quality of life. Unfortunately, very few studies have reported on the effects of EBMTs on GERD. In particular, there is no randomized controlled study (RCT) on the occurrence of GERD based on the type of procedure. This review aimed to investigate how EBMTs affect GERD.

## 2. Intragastric Balloon

An IGB is used extensively in clinical practice and is the earliest EBMT [16]. IGBs occupy space in the stomach and reduce food intake while simultaneously reducing gastric motility, which leads to weight loss. Various types of IGBs have been developed, and the most representative IGB is the BioEnteric intragastric balloon (Allergan, Irvine, CA, USA) [16]. The effectiveness of the IGB has been demonstrated in many studies. Six months of IGB treatment led to an average weight loss of 14.7 kg and a decrease of body mass index (BMI) by 5.7 kg/m^2^ [17]. Another systematic review showed that the mean changes in weight and BMI were 15.7 kg and 5.9 kg/m^2^, respectively, after 6 months [18].

Very few studies have investigated the effects of EBMT on GERD, and studies on IGBs are limited. In addition, some studies have reported conflicting results about how IGBs increase GERD. Tolone et al. demonstrated the effects of various bariatric procedures on lower esophageal sphincter pressure (LESp), peristalsis, and acid exposure total (AET) using high-resolution manometry and impedance–pH monitoring [19]. In that study, 13 patients underwent endoscopic balloon placement. As results, LESp and the frequency of ineffective peristalsis did not change significantly after placement of an IGB. AET and the total number of refluxes were also not significantly different before and after placement of an endoscopic balloon. In contrast, AET and the total number of refluxes increased after gastric banding and sleeve gastrectomy. Bariatric surgery transforms the stomach anatomy and induces physiological changes to accommodate internal pressure. These physiological changes create conditions in which GERD occurs. IGBs can be removed if necessary.

Several other studies have reported that IGBs cause transient or long-term GERD. Gastric distension is long thought to accelerate acid reflux because of reduced LESp and total sphincter length, and an increase in the frequency of transient LES relaxations (TLESR) and gastric acid secretion [20,21]. IGBs can also induce chronic artificial gastric distension and exacerbate GERD through the above mechanisms. However, weight loss occurs after balloon treatment, resulting in a pattern in which GERD-inducing factors due to obesity (increased abdominal pressure and TLESR) are cancelled out [22]. Therefore, the acid reflux parameters improve during the second half of IGB treatment compared to the value before IGB treatment [20]. Another study showed that chronic distention caused by an IGB increased acid reflux for 10 weeks after balloon placement, which resolved after 20 weeks [23].

Rossi et al. investigated the occurrence of erosive esophagitis after IGB treatment [24]. The proportion of patients affected by erosive esophagitis increased significantly after IGB treatment, although the difference was small [24]. Another systemic review demonstrated that GERD is a common complication experienced by patients treated with IGB (14.3%), but the proportion with esophagitis was relatively small (2.8%) [18]. Another study examined how IGB affects the DeMeester score [25]. In that study, LESp did not change significantly after IGB treatment, but the DeMeester score increased [25], suggesting that GERD caused by IGB treatment is not simply a change in LESp but is caused by other mechanisms. Placing an IGB may cause temporary changes in LESp and the physiology of the stomach, TLESR threshold, and regurgitation [26]. Another study reported how the position of the IGB affects the occurrence of GERD [27]. That study was performed on subjects divided into fundus and antral groups according to the position of the balloon. As a result, an IGB placed in the antrum led to a longer duration of GERD compared with one placed in the fundus [27].

Meta-analysis studies have shown that GERD occurs differently depending on the IGB type and filling volume [28,29]. A systematic review reported that GERD occured in subjects who received three of the four FDA-approved IGBs, such as the Orbera, ReShape Dual Balloon, and Obalon [29]. In another meta-analysis, the balloon was well-tolerated regardless of filling volume, with no difference in the rate of GERD. However, esophagitis was seen more frequently with lower filling volumes (<600 mL) [28]. The reason is unclear, but the location in the stomach varies depending on the weight of the balloon, which may change the degree of gastroesophageal reflux.

## 3. Endoscopic Sleeve Gastroplasty

ESG is a method to reduce stomach volume by endoscopic suturing [30]. RYGB and LSG are the most effective methods for weight loss, but they have a relatively high risk, and it is difficult to convert the stomach back to its original structure [31]. Fayad et al. demonstrated that ESG patients had a significantly lower rate of adverse events than LSG patients did (5.2% vs. 16.9%, *p* < 0.05) [32]. A recent meta-analysis also showed that adverse events with ESG totalled 2.9% and with LSG was 11.8% (*p* = 0.001) [33]. ESG narrows the intraluminal space by repeatedly suturing the greater curvature of the stomach body to the longitudinal axis using an endoscopic suturing system (OverStitch). Unlike LSG, ESG does not require general anesthesia and does not require gastric resection [30]. ESG requires additional equipment under moderate sedation (overtube and large-bore gastroscope are needed along with patient compliance to stitch), and in some cases may take longer than LSG. The ESG mechanism is to narrow the intraluminal space and to induce a feeling of early satiety, which ultimately leads to reduced food intake and weight loss. A prospective study reported that mean BMI loss was 7.3 ± 4.2 kg/m^2^, and a mean percentage of total body weight loss of 18.7 ± 10.7 after 1 year [34]. Another prospective study showed that ESG alters gastric physiology (slows gastric emptying and increases insulin sensitivity) and induces body weight loss in obese patients [35].

Very few studies have been performed on the effects of ESG on GERD, and the results are limited. In most cases, these studies compared the occurrence of adverse events with LSG. According to a recent meta-analysis, GERD with ESG produced a frequency of 0.4% adverse events (95% confidence interval (CI): 0.1–1.1) and LSG led to 5.8% adverse events (95% CI: 3.5–9.3) (*p* = 0.001) [33]. LSG can adversely affect GERD. A meta-analysis showed that the pooled incidence of new-onset GERD symptoms is 20% after LSG [36]. DuPree et al. demonstrated that LSG does not improve GERD symptoms in obese patients but, rather, may induce GERD in some previously asymptomatic patients [14]. Therefore, preoperative GERD is associated with a worse outcome after LSG. An expert consensus indicated that GERD is a contraindication to LSG [37]. The possible causes of LSG inducing GERD are low maximal distal contraction integral and low resting esophageal sphincter pressure [13,38]. Fayad et al. showed how ESG and LSG affect GERD [32]. In that study, the proportions of patients with GERD at baseline were similar in the two groups (ESG vs. LSG); however, new-onset GERD was significantly lower in the ESG group than in the LSG group (1.9% vs. 14.5%, *p* < 0.05) [32]. The reason why the frequency of GERD was very low in the ESG group is because the fundus of the stomach was preserved and the neuronal innervation of the stomach was maintained [39].

## 4. Conclusions

Although many studies have been published on EBMTs, studies showing the effect of EBMTs on GERD are rare. In addition, most of the published results simply present how the frequency of GERD was an adverse event after EBMT treatment. Table 1 indicates the effect of EBMT on the occurrence of GERD in previous studies. The reason why relatively few studies have been published on the effect of EBMT on the development of GERD is that the mechanism of EBMT-induced GERD is unclear, and the results of each study differ. In addition, countermeasures for more serious adverse events than GERD have been prioritized. Taken together, the following conclusions can be drawn. First, IGB may have some effects on the occurrence of GERD; however, the mechanism remains controversial. Gastric distension is induced by an IGB, and a decrease in LESp is thought to be the main reason for GERD. It is also expected that IGB will lower the TLESR threshold and induce regurgitation, creating an environment prone to GERD. Second, ESG has little effect on the occurrence of GERD. Far fewer studies have been published on ESG than on IGBs, and most do not mention GERD. In particular, we confirmed that the occurrence of GERD was much lower than that of LSG. Further prospective RCT studies with objective criteria (esophageal pH monitoring tests/endoscopy) are required to understand the effect of EBMTs on GERD.

## Figures and Tables

**Table 1:** Clinical outcomes of endoscopic bariatric and metabolic therapies for gastroesophageal reflux disease.

Author, Year	Study Design	Intervention	Total *N*	Patient Inclusion Criteria	Rate of GERD	Notes
Mathus-Vliegen et al., 2002 [20]	Randomized, double-blind, sham-controlled trial	IGB	43	BMI ≥ 32 kg/m^2^	NA	Supine reflux and duration of the longest reflux increased initially in balloon-treated subjects. However, acid reflux decreased to the pretreatment level during the second half of the treatment, and improved further after removing the balloon.
Sallet et al., 2004 [40]	Prospective, multicenter study	IGB	323	NA	Reflux esophagitis: 12.4%	
Al-Momen et al., 2005 [41]	Retrospective study	IGB	44	NA	GERD: 6.8%	
Herve et al., 2005 [42]	Prospective study	IGB	100	NA	New or progressive esophagitis: 7.5%	
Rossi et al., 2007 [24]	Retrospective study	IGB	121	BMI ≥ 30 kg/m^2^ with significant health risk; Patients with BMI ≥ 40 or 35 kg/m^2^ with co-morbidities; Presurgical temporary use in extremely obese patients	Erosive esophagitis: 15% (before treatment) → 18.2% (after treatment)	This study did not measure GERD but only showed an increase in erosive esophagitis.
Peker et al., 2010 [43]	Prospective study	IGB	31	NA	GERD: 16.1%	
Tai et al., 2013 [44]	NA	IGB	28	BMI: 27–32 kg/m^2^ with obesity-related co-morbidities; BMI ≥ 32 kg/m^2^ with obesity-related co-morbidities and did not wish to undergo bariatric surgery; BMI ≥ 37 kg/m^2^	Erosive esophagitis: 7.1% (before treatment) → 32.1% (after treatment), GERD: 7.1%	
Nguyen et al., 2017 [45]	Retrospective study	IGB	135	BMI ≥ 27 kg/m^2^	GERD: 6.7%	
Courcoulas et al., 2017 [46]	Multicenter, randomized, open-label clinical trial	IGB	160	BMI: 30–40 kg/m^2^	Severe GERD: 0.6%, esophagitis: 2.5%	
Dang et al., 2018 [47]	A propensity-matched analysis study	IGB	781	NA	GERD: 22.7%	The Metabolic and Bariatric Surgery Accreditation and Quality Improvement Program (MBSAQIP) collects data from 791 bariatric surgery centers in the United States and Canada.
Abeid et al., 2019 [48]	Interventional study	IGB	1600	NA	Reflux esophagitis: 3.6%	Most cases of reflux esophagitis were controlled by PPIs.
Barrichello et al., 2020 [25]	Retrospective review of prospectively collected data	IGB	24	BMI: 30–40 kg/m^2^	NA	There was an increase in the mean DeMeester score with the IGB treatment compared to pretreatment, without statistical significance.
Fayad et al., 2019 [32]	A case-matched study	ESG	54	NA	GERD: 1.9%	New-onset GERD was significantly lower in the ESG than in the LSG (1.9% vs. 14.5%, *p* < 0.05).
Fiorillo et al., 2020 [49]	Retrospective single-center study	ESG	23	BMI > 40 kg/m^2^ or BMI > 35 kg/m^2^ when diagnosed with obesity-related diseases	GERD: 0%	Contrast to the ESG group, 30.7% of the LSG group developed postoperative GERD.

GERD, gastroesophageal reflux disease; IGB, intragastric balloon; BMI, body mass index; NA, not available; PPI, proton pump inhibitor; ESG, endoscopic sleeve gastroplasty; LSG, laparoscopic sleeve gastrectomy.

## Data Availability

Not applicable.
